# α_2_-macroglobulin and α_1_-inhibitor-3 mRNA expression in the rat liver after slow interleukin-1 stimulation

**DOI:** 10.1155/S0962935196000385

**Published:** 1996-09

**Authors:** T. Gudmundsson, P. Björk, K. Ohlsson

**Affiliations:** 1 Department of Surgical Pathophysiology University of Lund Malmö University Hospital Malmö S-205 02 Sweden

## Abstract

In this study we have investigated total fiver RNA and the expression of mRNA in the rat fiver *in vivo* after a slow stimulation of interleukin-1. A total dose of 4 μg interleukin-1β was administered via a subcutaneously implanted osmotic minipump over a period of 7 days. Plasma concentrations of α_2_-macroglobulin manifested a rapid increase, reaching a peak on day 2, while α_1_-inhibitor-3 manifested a marked initial decrease to 50% of the baseline level, followed by a tendency to increase again. For measurement of total RNA and specific mRNAs from the fiver, rats were sacrificed at different times during the experimental period. Total RNA peaked at 6 h, the level being approximately 60% higher than baseline value. Specific mRNA from the liver for α_2_-macroglobulin and α_1_-inhibitor-3 were quantified using laser densitometry on slot blots. The amounts measured during the experimental period agreed with the pattern of corresponding plasma protein levels. From barely detectable amounts at baseline, α_2_-macroglobulin mRNA peaked on day 1, and then declined. Levels of α_1_-inhibitor-3 mRNA manifested an initial increase at 3 h, but then declined and remained low until day 5 when there was a tendency towards an increase. It was concluded that the levels of plasma concentrations of α_2_-macroglobulin and α_1_-inhibitor-3 are mainly regulated at the protein synthesis level, and that long-term interleukin-1β release could not override the initial acute phase protein counteracting mechanism triggered.


Mediators of Inflammation 5, 266–270 (1996)

### $\alpha_2$-macroglobulin and $\alpha_1$-inhibitor-3 mRNA expression in the rat liver after slow interleukin-1 stimulation

T. Gudmundsson[CA], P. Björk and K. Ohlsson

Department of Surgical Pathophysiology, University of Lund, Malmö University Hospital, S-205 02 Malmö, Sweden

[CA]Corresponding Author

**IN this study we have investigated total liver RNA and the expression of mRNA in the rat liver *in vivo* after a slow stimulation of interleukin-1. A total dose of 4 μg interleukin-1β was administered via a subcutaneously implanted osmotic mini-pump over a period of 7 days. Plasma concentrations of $\alpha_2$-macroglobulin manifested a rapid increase, reaching a peak on day 2, while $\alpha_1$-inhibitor-3 manifested a marked initial decrease to 50% of the baseline level, followed by a tendency to increase again. For measurement of total RNA and specific mRNAs from the liver, rats were sacrificed at different times during the experimental period. Total RNA peaked at 6 h, the level being approximately 60% higher than baseline value. Specific mRNA from the liver for $\alpha_2$-macroglobulin and $\alpha_1$-inhibitor-3 were quantified using laser densitometry on slot blots. The amounts measured during the experimental period agreed with the pattern of corresponding plasma protein levels. From barely detectable amounts at baseline, $\alpha_2$-macroglobulin mRNA peaked on day 1, and then declined. Levels of $\alpha_1$-inhibitor-3 mRNA manifested an initial increase at 3 h, but then declined and remained low until day 5 when there was a tendency towards an increase. It was concluded that the levels of plasma concentrations of $\alpha_2$-macroglobulin and $\alpha_1$-inhibitor-3 are mainly regulated at the protein synthesis level, and that long-term interleukin-1β release could not override the initial acute phase protein counteracting mechanism triggered.**

**Key words:** $\alpha_1$-inhibitor-3, $\alpha_2$-macroglobulin, Interleukin-1, mRNA, Osmotic mini-pump, Proteinase inhibitors, RNA

### Introduction

Different tissue injuries, such as those due to trauma, radiation, infection and neoplasia, cause an inflammatory reaction involving the complex co-ordination of a variety of cells and inflammatory mediators. One of these mediators, the cytokine interleukin-1 (IL-1),[1] is crucially involved in this complex series of events. IL-1 is produced by many different cells, but predominantly by monocytes and macrophages. It has the capability of inducing an acute phase protein response which differs from species to species.[2] In the rat, $\alpha_2$-macroglobulin ($\alpha_2$M), synonymous with $\alpha_2$-acute phase protein,[3] is a positive acute phase protein, while $\alpha_1$-inhibitor-3 ($\alpha_1I_3$) is a negative acute phase protein.[4] They are both proteinase inhibitors belonging to the thiol ester protein family. Their amino acid sequences have been determined.[5,6] When rats are subjected to an experimentally induced inflammatory reaction, or when IL-1 is administered *in vivo*, the plasma concentration of $\alpha_2$M increases but that of $\alpha_1I_3$ decreases.[3,4,7] The expression of mRNA for both proteins has earlier been investigated in experimental inflammatory reactions, $\alpha_2$M having been shown to be characterized by a marked increase in mRNA expression[5] but $\alpha_1I_3$ by a decrease.[6,8] In a recent study we showed that the initial changes in the plasma concentrations of $\alpha_2$M and $\alpha_1I_3$ could not be preserved by a slow continuous stimulation of IL-1.[9]

The aim of the present study was to compare total liver RNA and the expression of $\alpha_2$M and $\alpha_1I_3$ mRNA in the liver after slow continuous IL-1 stimulation *in vivo* with the corresponding plasma levels.

### Materials and Methods

*Animal experiment:* Female Wistar rats (Mölle-gaard Avelslaboratorium A/S, Skensved,

© 1996 Rapid Science Publishers

Denmark) weighing 210–250 g were used. After the animals had been anaesthetized with an intraperitoneal injection of Mebumal®, their backs were shaved and disinfected. Through a small incision in the skin of approximately 15 mm, an osmotic mini pump (2001, Alzet®) was implanted subcutaneously and the wound was closed with Michel® clips. The flowrate of the pump was 1 µl/h. After implantation of the pumps, the rats were allowed to wake up. Two rats that received pumps without IL-1 served as controls, and 24 animals received pumps containing 4 µg human recombinant IL-1β (Synergen Inc., USA). The protein was diluted to appropriate concentration in sterile phosphate buffered saline, pH 7.4 (Dulbecco), with 0.2% (w/v) bovine serum albumin (Sigma), before being used to fill the pumps. Blood samples (0.4 ml) was collected from the tails into EDTA containing tubes and plasma was immediately prepared and frozen at –70°C until analysed. Samples were taken at 0 h, 3 h, 6 h, 24 h, 3 days, 5 days and 7 days after implantation of the pumps. Two animals were killed with an overdose of Mebumal® after 3 and 6 h, respectively, and four animals were killed after 24 h, 2, 5 and 7 days, respectively. Liver biopsies were taken under sterile conditions and frozen at –70°C until analysed. Liver biopsies from two normal unstimulated rats provided baseline reference levels at time 0.

During the whole experimental period the rats had free access to water and standard pellets. The animal experiments were sanctioned by the local ethics committee for animal experiments.

*Assays:* Rat $\alpha_2M$ and $\alpha_1I_3$ were measured by electroimmunoassay (EIA).[10] Antisera against $\alpha_2M$ and $\alpha_1I_3$ were prepared as described previously.[3,4] In order to measure IL-1 in the rat plasma a specific biotinylated polyclonal antibody against human recombinant IL-1β was used in an enzyme-linked immunosorbent assay. The sensitivity of the assay was 0.15 µg/l.

*RNA analysis:* RNA from the liver biopsies was prepared with the guanidine HCl method[11] and stored as an ethanol precipitate at –70°C prior to use. Four single-stranded 30–34-mer oligonucleotides for rats $\alpha_2M$ and $\alpha_1I_3$, respectively, were produced by British Biotechnology Products Ltd (Oxon, UK). The probes were modified at the 5′ end by the addition of alkaline phosphatase. They were used in the form of a cocktail of an equimolar mixture of the probes for each protein. The slot blot procedure was performed as described previously.[12] Five µg of mRNA was analysed. A β-actin probe was used as internal standard. Electrophoresis of RNA was performed on 1% agarose gels containing 6% formaldehyde while RNA transfer and filter hybridization was performed as described by Bond and Farmer.[13] The probes labelled with alkaline phosphatase were detected with Lumigen TM PPD, according to the manufacturer's instructions (Boehringer Mannheim GmbH Biotechnica, Mannheim, Germany).

*Laser densitometry:* Relative quantification of slot blots was obtained from appropriately exposed photographic films using a LKB Ultroscan XL laser densitometer (Uppsala, Sweden).

### Results

No rats manifested any discomfort at any time during the experiments.

*Tissue reactions:* Macroscopic cystic formation with fluid was present around the pumps in rats from day 2 and throughout the remainder of the experimental period.

*Plasma IL-1 levels:* In controls and in all plasma samples at 0 h, 3 h and 6 h, IL-1 concentrations fell below the sensitivity for the assay used while in rats with IL-1 containing mini-pumps, plasma concentrations ranged between 0.15 and 0.44 µg/l in samples from 24 h to 7 days.

*Acute phase proteins:* Plasma $\alpha_2M$ and $\alpha_1I_3$ concentrations were measured by EIA (Fig. 1). In IL-1 stimulated rats plasma $\alpha_2M$ concentrations were undetectable at time 0, but then increased reaching a peak at 2 days, after which they declined during the remainder of the experimental period. From their peak level at time 0, plasma concentrations of $\alpha_1I_3$ gradually decreased to a nadir at day 3, after which they increased slightly. In the controls, a slight increase was seen in $\alpha_2M$ levels with a peak at 24 h and there was a minor decrease in $\alpha_1I_3$ levels which persisted throughout the experimental period.

*Total RNA:* At 6 h total RNA increased reaching a level approximately 60% greater than the initial value (Fig. 2). From days 1 to 7 levels were approximately 20–25% greater than the initial value.

*mRNA detection:* Slot blots were hybridised with probes for $\alpha_2M$ and $\alpha_1I_3$ mRNA, and were then analysed with laser densitometry. $\alpha_2M$ mRNA was barely detectable at time 0 but manifested a clear increase at 3 h, after which it continued to

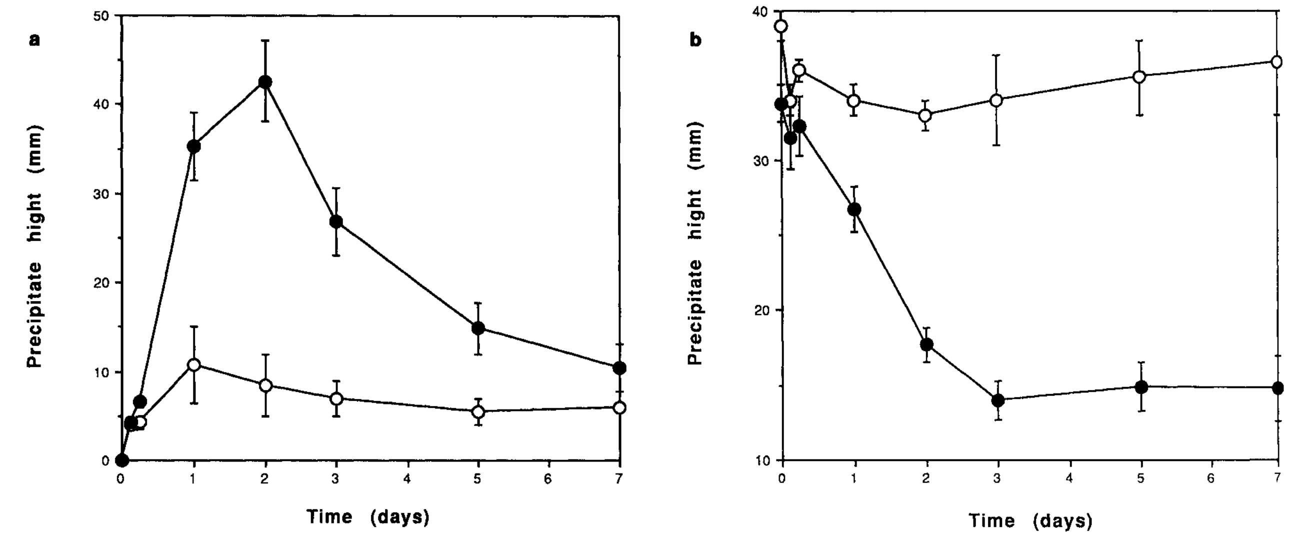

FIG. 1. Plasma levels of $\alpha_2M$ (a) and $\alpha_1I_3$ (b) measured by EIA, for IL-1-stimulated animals (●) and controls (○). Osmotic mini-pumps containing IL-1 (4 μg) were implanted subcutaneously at time 0 and IL-1 was released slowly over a period of 7 days. Controls ($n$ = 2) received mini-pumps containing vehicle only. To obtain liver biopsies for RNA analysis rats were sacrificed successively, and thus the number of observations decreases over time ($n$ = 26 at 0, $n$ = 24 at 3 h, $n$ = 22 at 6 h, $n$ = 20 at 1 day, $n$ = 16 at 2 days, $n$ = 12 at 3 days, $n$ = 8 at 5 days and $n$ = 4 at 7 days). Values are given as means ± standard error of the mean.

increase reaching a peak at 24 h (Fig. 3), before declining toward the starting level. $\alpha_1I_3$ mRNA manifested an initial increase at time 3 h, but then decreased reaching a nadir at days 1–3, after which it tended to increase again (Fig. 3).

### Discussion

A variety of different cells and inflammatory mediators interact in a complex way to overcome and heal a tissue injury. IL-1 is one of the most potent cell activators in this complex series of interactions.[1] It has the capacity to mediate a variety of responses such as fever, slow wave sleep, acute phase response and anorexia. The two forms of IL-1, α and β, both act on the IL-1 receptor of which there are also two different types, called IL-1 receptors I and II.[14] Finally, there is also a true receptor antagonist called IL-1 receptor antagonist.[15] This triad consisting of IL-1, the IL-1 receptors and the IL-1 receptor antagonist (IL-1ra) is crucially involved in the inflammatory reaction. An illustration of this is the fact that a lethal endotoxin shock in rabbits may be remedied by the administration of human recombinant IL-1ra.[16]

In this work we have studied the effects of a slow continuous IL-1 stimulation upon total liver RNA, liver mRNA and plasma concentrations of $\alpha_2M$ and $\alpha_1I_3$ in the rat. Four mg IL-1 was administered over a period of 7 days. The mini-pumps were found to deliver IL-1 continuously. Detectable concentrations (0.15–0.44 μg/l) of IL-1 could be demonstrated in plasma from IL-1 stimulated rats during the period of 24 h to 7 days while no detectable concentrations of IL-1 could be demonstrated in samples from 0 to 6 h.

Rat $\alpha_2M$ and $\alpha_1I_3$ are both protease inhibitors, and they are also acute phase proteins.[17] The human and the rat protein manifest an amino acid sequence homology of 73%.[5] Although $\alpha_2M$ is not an acute phase reactant in humans, in the rat it is both a fast and strong positive acute phase reactant. Its function in humans is considered to be solely that of a proteinase inhibitor.

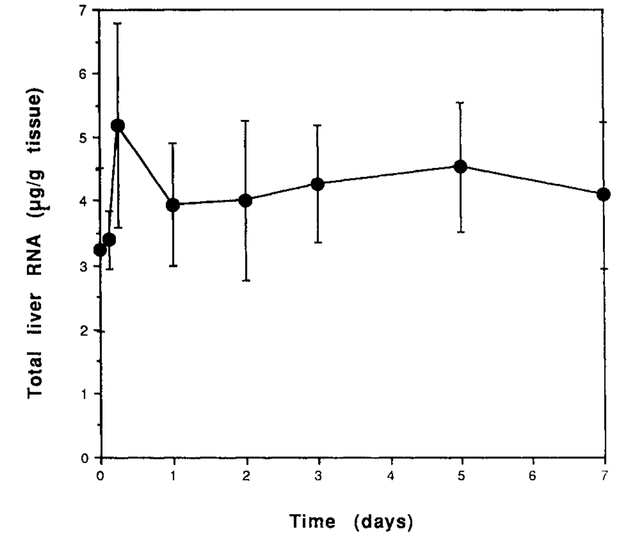

FIG. 2. Total RNA determined with the guanidine HCl method. Rats were stimulated with a slow IL-1 release from an osmotic mini-pump subcutaneously implanted. To obtain liver biopsies rats were sacrificed subsequently ($n$ = 2 at 0, $n$ = 2 at 3 h, $n$ = 2 at 6 h, $n$ = 4 at 1 day, $n$ = 4 at 2 days, $n$ = 4 at 3 days, $n$ = 4 at 5 days and $n$ = 4 at 7 days). Values are given as means ± standard error of the mean.

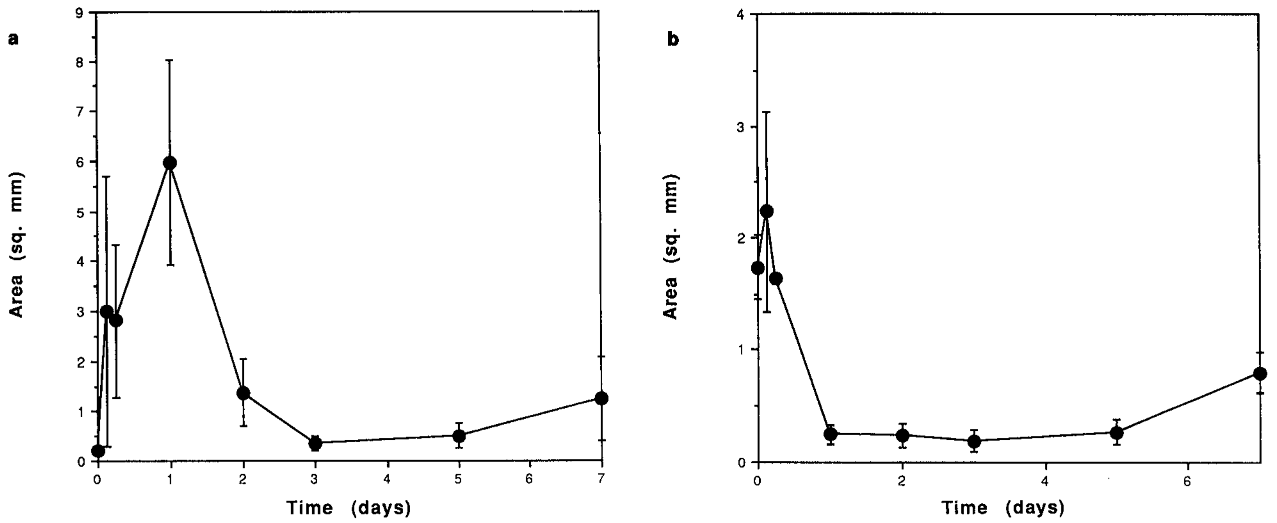

FIG. 3. mRNA for $\alpha_2M$ (a) and $\alpha_1I_3$ (b) measured in continuously IL-1-stimulated rats. Hybridized slot blots were analysed by laser densitometry for relative quantification by measuring the area. Values are given as means $\pm$ standard error of the mean. To obtain liver biopsies rats were sacrificed successively ($n = 2$ at 0, $n = 2$ at 3 h, $n = 2$ at 6 h, $n = 4$ at 1 day, $n = 4$ at 2 days, $n = 4$ at 3 days, $n = 4$ at 5 days and $n = 4$ at 7 days).

On the other hand, rat $\alpha_1I_3$ has no counterpart in man. In the rat it is one of the most abundant plasma proteins with a normal concentration of 6–10 g/l. The protein is a proteinase inhibitor, but also manifests binding capacity for other proteins such as rat $\alpha_1$-microglobulin[18] which suggests it to have other complementary functions. $\alpha_1I_3$ clones are capable of binding to polyclonal specific antisera, as was shown when a rat liver λgt11 cDNA library was screened for other proteins.[8,19]

We have induced an acute phase protein response by a slow continuous stimulation of IL-1 delivered by an osmotic mini-pump implanted subcutaneously. Plasma $\alpha_2M$ concentrations, as measured by EIA, manifested a rapid increase starting at 3 h and reaching a maximum at 2 days. A striking feature was the rapid decrease of this plasma concentration once the peak had been reached. The results of laser densitometry of $\alpha_2M$ mRNA on slot blots from rat livers manifested a similar pattern, though the peak occurred on day 1 instead. The longer time taken for the plasma concentrations to peak is consistent with the extra time required for protein synthesis. The occurrence of the $\alpha_2M$ mRNA peak on day 1 (24 h) is consistent with findings in a previous study by Gehring and co-workers,[5] where the level of $\alpha_2M$ mRNA reached a peak 20 h after induction of an experimental inflammatory reaction in the rat. Hepatic acute phase genes have been divided into two classes according to the cytokines that are their main inducers.[20] Class 1 genes are regulated by both IL-1 and IL-6, while class 2 genes are regulated by IL-6 type genes. As the rat $\alpha_2M$ gene belongs to class 2 it is not induced by IL-1. The $\alpha_2M$ acute phase response observed after IL-1 stimulation in this study was thus probably induced by IL-6 released by IL-1. Plasma $\alpha_1I_3$ concentrations manifested an early decrease after the start of the IL-1 stimulation, the nadir occurring at day 3. The levels of $\alpha_1I_3$ mRNA first manifested an early increase at 3 h, and were then decreased sevenfold at days 1–5, but at day 7 again manifested a tendency to increase. The initial increase is in agreement with findings in a study by Aiello and co-workers[8] who noted a 25% increase in $\alpha_1I_3$ mRNA 6 h after induction of an experimental inflammatory reaction in the rat.

When IL-1 is administered *in vivo* in a slow release manner, it is not possible to maintain the increased $\alpha_2M$ plasma levels. Therefore some counteracting mechanism must be triggered by the initial IL-1 stimulation. Different mechanisms might be suggested. IL-1 has the capacity of inducing IL-6 production,[21] which in turn has been shown to induce IL-1ra production *in vivo*.[22] In addition, monocytes have also been shown to produce IL-1ra when stimulated by different acute phase proteins like C-reactive protein and $\alpha_1$-antitrypsin.[23] Another possible mechanism is the up-regulation of the IL-1 type II receptor in polymorphonuclear leukocytes, mediated by IL-13, which may act as a 'decoy' receptor for IL-1.[24,25] However, it remains unknown whether IL-1 can induce production of IL-13 *in vivo*.

Total RNA in the liver manifested a marked increase noted at 6 h after the start of the IL-1 stimulation, after which it decreased to a level approximately 20–25% greater than the initial level. The increase of total liver RNA is in agreement with the increase seen after turpentineinduced inflammation in the rat.[26] The persistence of increased levels of total liver RNA may be interpreted as a consequence of a slower course due to the huge numbers of proteins being produced by the liver, rather than a consequence of a long-term stimulation by IL-1.

To sum up, we have found slow continuous exposure to IL-1 to induce an acute phase protein response in $\alpha_2$M and $\alpha_1I_3$, characterized by changes in plasma concentrations and corresponding changes in liver mRNA levels. Although the IL-1 stimulation continued for 7 days, it was not possible to maintain the acute phase response, which suggests the presence of a counteracting mechanism triggered initially.

### References

1. Dinarello CA. Interleukin-1 and is biologically related cytokines. *Adv Immunol* 1989; **44:** 153–205.
2. Koj A, Magielska-Zero D, Kurdowska A, Bereta J. Proteinase inhibitors as acute phase reactants: regulation of synthesis and turnover. *Adv Exp Med Biol* 1988; **240:** 171–181.
3. Gauthier F, Mouray H. Rat $\alpha_2$ acute phase macroglobulin isolation and physicochemical properties. *Biochem J* 1976; **159:** 661–665.
4. Gautheir F, Ohlsson K. Isolation and some properties of a new enzyme-binding protein in rat plasma. *Hoppe-Seyler's Z Physiol Chem* 1978; **359:** 387–992.
5. Gehring MR, Shiels BR, Northemann W, Bruijn MHL, Kan CC, Chain AC, Noonan DJ, Fey GH. Sequence of rat liver α-macroglobulin and acute phase control of its messenger RNA. *J Biol Chem* 1987; **262:** 446–454.
6. Braciak TA, Northemann W, Hudson GO, Shields BR, Gehring MR, Fey GH. Sequence and acute phase regulation of rat $\alpha_1$-inhibitor III messenger RNA. *J BIol Chem* 1988; **263:** 3999–4012.
7. Björk P, Ohlsson K. The interleukin-1 receptor antagonist influences interleukin-1 effects in rat and mouse. *Med Infl* 1992; **1:** 27–31.
8. Aiello LP, Shia MA, Robinson GS, Pilch PF, Farmer SR. Characterization and hepatic expression of rat $\alpha_1$-inhibitor III mRNA. *J Biol Chem* 1988; **263:** 4013–4022.
9. Björk P, Gudmundsson T, Ohlsson K. Acute phase protein response and polymorphonuclear leukocyte cathepsin G release after slow interleukin-1 stimulation in the rat. *Med Infl* 1994; **3:** 425–431.
10. Laurell CB. Electroimmuno assay. *Scand J Clin Lab Invest* 1972; **29** (suppl 124): 21–37.
11. Strohman RC, Moss PS, Micou-Eastwood J, Spector D, Przybyla A, Paterson B. Messenger RNA for myosin polypeptides: isolation from single myogenic cell cultures. *Cell* 1977; **10:** 265–273.
12. Dyson NJ. Immobilization of nucleic acids and hybridization analysis. In: Brown TA, ed. *Essential Molecular Biology: A Practical Approach. Vol. 2.* Oxford: IRL Press, 1991; 111–156.
13. Bond JF, Farmer SR. Regulation of tubulin and actin mRNA production in rat brain: expression of a new beta-tubulin mRNA with development. *Mol Cell Biol* 1983; **3:** 1333–1342.
14. Granowith EV, Clark BD, Mancilla J, *et al.* Interleukin-1 receptor antagonist competitively inhibits the binding of interleukin-1 to the type II interleukin-1 receptor. *J Biol Chem* 1991; **266:** 14147–14150.
15. Hannum CH, Wilcox CJ, Arend WP, *et al.* Interleukin-1 receptor antagonist activity of a human interleukin-1 inhibitor. *Nature* 1990; **343:** 336–340.
16. Ohlsson K, Björk P, Bergenfeldt M, Hageman R, Thompson RC. Interleukin-1 receptor antagonist reduces mortality from endotoxin shock. *Nature* 1990; **348:** 550–552.
17. Loneberg-Holm K, Reed DL, Roberts RC, Herbert RR, Hillman MC, Kutney RM. Three high molecular weigh protease inhibitors of rat plasma. *J Biol Chem* 1987; **265:** 438–445.
18. Falkenberg C, Grubb A, Åkerström B. Isolation of rat $\alpha_1$-microglobulin. *J BIo Chem* 1990; **265:** 16150–16157.
19. Schweizer M, Takabayashi K, Geiger T, Laux T, Biermann G, Buhler JM, Gauthier F, Roberts LM, Heinrich PC. Identification and sequencing of cDNA clones for the rodent negative acute-phase protein alpha 1-inhibitor 3. *Eur J Biochem* 1987; **164:** 375–381.
20. Baumann H, Gauldi J. Regulation of hepatic acute phase plasma protein genes by hepatocyte stimulating factors and other mediators of inflammation. *Mol Biol Med* 1990; **7:** 147–159.
21. Zoja C, Wang JM, Bettoni S, *et al.* Interleukin-1β and tumor necrosis factor-α induce gene expression and production of leukocyte chemotactic factors, colony stimulating factors, and interleukin-6 in human mesangial cells. *Am J Pathol* 1991; **138:** 991–1003.
22. Tilg H, Trehu E, Atkins MB, Dinarello CA, Mier JW. Interleukin-6 (IL-6) as an anti-inflammatory cytokine: induction of circulating IL-1 receptor antagonist and soluble tumor necrosis factor receptor p55. *Blood* 1994; **83:** 113–118.
23. Tilg H, Vannier E, Vachino G, Dinarello CA, Mier JW. Antiinflammatory properties of hepatic acute phase proteins: preferential induction of interleukin 1 (IL-1) receptor antagonist over IL-1 beta synthesis by human peripheral blood mononuclear cells. *J Exp Med* 1993; **178:** 11629–11636.
24. Coletta F, Re F, Muzio M, Polentarutti N, Minty A, Caput D, Ferrara P, Mantovani A. Interleukin-13 induces expression and release of interleukin-1 decoy receptor in human polymorphonuclear cells. *J Biol Chem* 1994; **269:** 12403–12406.
25. Coletta F, Dower SK, Sims JE, Mantovani A. The type II 'decoy' receptor: a novel regulatory pathway for interleukin 1. *Immunol Today* 1994; **15:** 562–566.
26. Demczuk S, Chandler AM. Changes in hepatic RNA, poly(A)+RNA and poly(A)–RNA during the acute phase response to inflammation. *Biochem Int* 1986; **12:** 155–165.

ACKNOWLEDGEMENTS. We wish to thank Iréna Ljungkrantz for excellent technical assistance and Dr R. C. Thompson, Synergen Inc., for providing human recombinant interleukin-1β. This work was supported by research funds from the Swedish Medical Research Council (project no. 3910), the Medical Faculty, University of Lund, the Swedish Medical Society; the Albert Påhlsson Foundation and the Foundations for Cancer and Medical Research administered by the Malmö University Hospital.

**Received 20 May 1996; accepted with revision 25 June 1996**
